# Nasopharyngeal and Adenoid Colonization by *Haemophilus influenzae* and *Haemophilus parainfluenzae* in Children Undergoing Adenoidectomy and the Ability of Bacterial Isolates to Biofilm Production

**DOI:** 10.1097/MD.0000000000000799

**Published:** 2015-05-08

**Authors:** Urszula Kosikowska, Izabela Korona-Głowniak, Artur Niedzielski, Anna Malm

**Affiliations:** From the Department of Pharmaceutical Microbiology With Laboratory for Microbiological Diagnostics, Medical University of Lublin (UK, IK-G, AM); Otoneurology Laboratory of III Chair of Pediatrics, Medical University of Lublin (AN), Lublin, Poland.

## Abstract

Haemophili are pathogenic or opportunistic bacteria often colonizing the upper respiratory tract mucosa. The prevalence of *Haemophilus influenzae* (with serotypes distribution), and *H. parainfluenzae* in the nasopharynx and/or the adenoid core in children with recurrent pharyngotonsillitis undergoing adenoidectomy was assessed. Haemophili isolates were investigated for their ability to biofilm production.

Nasopharyngeal swabs and the adenoid core were collected from 164 children who underwent adenoidectomy (2–5 years old). Bacteria were identified by the standard methods. Serotyping of *H. influenzae* was performed using polyclonal and monoclonal antisera. Biofilm formation was detected spectrophotometrically using 96-well microplates and 0.1% crystal violet.

Ninety seven percent (159/164) children who underwent adenoidectomy were colonized by *Haemophilus* spp. The adenoid core was colonized in 99.4% (158/159) children, whereas the nasopharynx in 47.2% (75/159) children (*P* < 0.0001). In 32% (51/159) children only encapsulated (typeable) isolates of *H. influenzae* were identified, in 22.6% (36/159) children only (nonencapsulated) *H. influenzae* NTHi (nonencapsulated) isolates were present, whereas 7.5% (12/159) children were colonized by both types. 14.5% (23/159) children were colonized by untypeable (rough) *H. influenzae*. In 22% (35/159) children *H. influenzae* serotype d was isolated. Totally, 192 isolates of *H. influenzae*, 96 isolates of *H. parainfluenzae* and 14 isolates of other *Haemophilus* spp. were selected. In 20.1% (32/159) children 2 or 3 phenotypically different isolates of the same species (*H. influenzae* or *H. parainfluenzae*) or serotypes (*H*. *influenzae*) were identified in 1 child. 67.2% (129/192) isolates of *H*. *influenzae,* 56.3% (54/96) isolates of *H. parainfluenzae* and 85.7% (12/14) isolates of other *Haemophilus* spp. were positive for biofilm production. Statistically significant differences (*P* = 0.0029) among *H. parainfluenzae* biofilmproducers and nonproducers in the adenoid core and the nasopharynx were detected.

*H*. *influenzae* and *H*. *parainfluenzae* carriage rate was comparatively higher in the adenoid core than that in the nasopharynx in children undergoing adenoidectomy, suggesting that their involvement in chronic adenoiditis. The growth in the biofilm seems to be an important feature of haemophili colonizing the upper respiratory tract responsible for their persistence.

## INTRODUCTION

Haemophili can exist as microbiota of the human upper respiratory tract mucosa.^1–3^*H*. *influenzae* and occasionally *H. parainfluenzae* are able to cause a variety of infections. The encapsulate strains of *H. influenzae* (mainly serotype b—Hib, but also serotypes a, c—f) are important in the pathogenesis of invasive infections and chronic or recurrent diseases that are most often reported in children and rarely in adults.^4–6^ For a long time Hib was considered as a major pathogen of young children. Since the introduction of Hib vaccines, invasive *H. influenzae* disease has been significantly eliminated.^7–9^ The increasing number of reports about other than Hib serotypes (mainly a, e, and f serotypes), which may be responsible for diseases, are spreading globally.^4,10–13^ Today it is known that *H. influenzae* without capsular polysaccharides, referred to as nontypeable (nonencapsulated) *H. influenzae* (NTHi), have also been associated with chronic or invasive diseases.^14–16^ NTHi remain important as causes of respiratory infections and are frequently associated with otitis media, chronic bronchitis, and community-acquired pneumonia. *H. parainfluenzae* is rarely considered as a documented etiologic agent for infectious diseases.^17–19^ However, this opportunistic species may cause systemic infections in terms of their course and symptoms resembling infections caused by NTHi, such as epiglottitis,^20^ meningitis,^21^ bacteremia or sepsis,^22,23^ bronchitis and chronic obstructive pulmonary disease or other respiratory infections.^24–26^*H. parainfluenzae* is also a part of HACEK group of gram-negative bacteria: *Haemophilus* species (*Haemophilus parainfluenzae*, *Haemophilus aphrophilus*, *Haemophilus paraphrophilus*), *Actinobacillus actinomycetemcomitans*, *Cardiobacterium hominis*, *Eikenella corrodens*, *Kingella* species, which may cause infective endocarditis.^27–29^

Biofilm is a structural community of microbials that is adherent to natural or synthetic surfaces.^30^ The properties of microorganisms, including those colonizing the upper respiratory tract, such as adherence and biofilm formation can foster the emergence of recurrent infection and failure of antimicrobials to eradicate the pathogens.^31,32^ According to the Center for Disease Control and Prevention, biofilm is estimated to be involved in at least 65% of human bacterial infections.^33^

Microbial colonization of the upper respiratory tract mucosa may be regarded as an important factor in the most common childhood diseases development.^34,35^ The problems of children are often associated with recurrent and chronic adenotonsillar infections, which may require surgical intervention. During the present studies, the prevalence of *H. influenzae* and *H. parainfluenzae* in samples cultured from the nasopharynx and the adenoid core of young children undergoing adenoidectomy and the ability of the haemophili isolates to biofilm production were assessed.

## MATERIALS AND METHODS

### Patients

Samples were collected from 164 children (Male—101, Female—63, ages 2–5 years old) who had undergone adenoidectomy for recurrent pharyngotonsilitis at the Department of Pediatric Otolaryngology, Phoniatry and Audiology, Medical University of Lublin, Poland, in May-June (second quarter) and November-December (fourth quarter) 2011. The indication for adenoidectomy was acute pharyngotonsillitis lasting for at least 2 years, with 5 or more acute attacks per year. Patients did not receive antimicrobial therapy for at least 20 days before the surgery. Patients scheduled for surgery were qualified by the pediatrician, otolaryngologist, and anesthesiologist. All patients had blood count, urinalysis, coagulation and blood type indicated. In the absence of variations in laboratory tests and auscultatory changes in the lung fields, all patients were anesthetized and operated. During surgery and in the postoperative period of at least 24 hours after completion of surgery, there were no significant complications. In 1 case there was late postoperative bleeding. In this case Foley catheter balloon tamponade was used.

In some studies, a group of 68 healthy children ages 2 to 5 years old (male—32, female—37) was also included. Children of this group did not experience recurrent respiratory tract infections and had not been admitted to hospital for at least 2 years but had mild nasopharyngeal infections seasonally.

Since 2007, all children in Poland had been subject to mandatory vaccination against Hib based on the calendar of vaccination. In each case, informed consent was obtained from the parents. The study design and protocol were approved by the Ethics Committee of Medical University of Lublin (No. KE-0254/75/2011).

### Sample Collection

A total of 328 samples (164 nasopharyngeal specimens obtained before adenoidectomy and 164 the adenoids after the surgery) were collected from children undergoing adenoidectomy. The nasopharyngeal specimens obtained before adenoidectomy and the adenoids after the surgery were cultured for haemophili. The samples were collected in sterile alginate-tipped swabs on aluminum shafts. The surgeon removed the adenoids through the mouth by making several small incisions and cauterized the site once the adenoids were removed. Antiseptic and/or other antimicrobial were not used during the surgery. After the surgery, the adenoids were placed in a sterile container. Then the samples were transferred during 2 hours to the Department of Pharmaceutical Microbiology and prepared according to Korona-Głowniak et al.^36^ One surface of the adenoid was cauterized with a heated scalpel and an incision was made through that cauterized area with a sterile scalpel, cutting the adenoid in half. The core was swabbed with sterile alginate-tipped applicator and inoculated on agar medium. In addition, 68 nasopharyngeal specimens were collected from healthy children.

### Bacterial Growth and Identification

The HAEM agar (Haemophilus chocolate agar; bioMérieux, Paris, France) with PolyVitex and haemoglobin or TSB+HTMS - TSB (Tripticase in Soy Broth; Biocorp, Warsaw, Poland) medium supplemented with HTMS (Haemophilus Test Medium Supplement; HTMS SRO158E, Oxoid, UK) with growth factors for haemophili (25 μg mL^−1^ of NAD and 15 μg mL^−1^ of hematin) were used. The samples were cultured on HAEM plates and then incubated for 18 to 24 hours at 35^o^C in approximately 5% CO_2_ atmosphere. After incubation, haemophili were identified using microscopic methods as gram-negative cocco-bacilli. For the initial identification of the isolated hemophili, morphological characteristics of the colonies growing on HAEM agar and requirements for hemin (X factor) and nicotinamide adenine dinucleotide (V factor) on TSA (Tripticasein Soy LAB-AGAR, Biocorp) medium with diagnostic Oxoid discs DD3 (X factor), DD4 (V factor), DD5 (both X and V factors) were determined. The colonies with morphological differences in relation to their size, opacity, roughness and color were initially classified as different strains and identified independently. Biochemical identification of isolates was carried out using the API NH system (bioMérieux). Species other than the *H. influenzae* or *H. parainfluenzae* were determined as *Haemophilus* spp. on the basis of API NH results. The bacterial isolates in TSB+HTMS medium with addition of 30% glycerol were stored at −70°C and utilized in further studies.

### Beta-Lactamase Detection

All haemophili isolates were tested for the production of beta-lactamase by *Pen* microtestusing API NH system and the results were compared with the cefinase test (BBL Cefinase β-Lactamase Detection Discs; BD BBL-Becton Dickinson and Company, Sparks, MD) impregnated with the chromogenic cephalosporin, nitrocefin.

### Serotyping

*H. influenzae* isolates (selected from smooth colonies) were serotyped by slide agglutination using Difco *Haemophilus Influenzae* Antisera (BD BBL-Becton, Dickinson and Company, Sparks, MD, USA) according to the manufacturer's procedure. Determinations were made as follows: 10 μL of polyvalent sera (containing all antibodies against all serotypes) was added to a glass slides and a macroscopically chosen isolate was added. The slide was rocked gently for 1 minute to mix the serum and bacteria. Strains with a positive reaction were further characterized successively against monovalent sera for the individual serotypes using the same technique. The results were determined on the basis of the intensity of the agglutination according to the instructions of the manufacturer (bioMérieux) as: 4+ (100% agglutination, background clear to slightly turbid), 3+ (75% agglutination, background slightly cloudy), 2+ (50% agglutination, background moderately turbid), 1+ (25% agglutination, background cloudy) and (−) as no agglutination. Among the tested strains agglutination which after 1 minute was ≥3+ was interpreted as a positive result.

Positive agglutination using polyserum and 1 of the individual typing sera confirms the serotype of *H*. *influenzae*. The isolates negative to the employed pooled sera, but positive to the polyserum were defined as nontypeable (nonencapsulated) *H*. *influenzae* (NTHi). Rough (R, untypeable) culture isolates (autoagglutination in sterile 0.85% saline) were not tested.

### Microtiter-Plate Biofilm Detection

Before each experiment, bacterial strains were subcultured on HAEM medium and incubated at 35^o^C in about 5% CO_2_ atmosphere. Overnight the cultures of all isolates were diluted in fresh TSB+HTMS medium and standardized with an initial optical density of at wavelength λ = 570 (OD_570_) = 0.08 ± 0.02 using a microplate reader (ELx800, BioTek, Instruments, Winooski, VT, USA).

The haemophili isolates were tested for biofilm production using 96-well polystyrene microplates (96F**-**Well Microplates Nunclon Delta Surface, Thermo Scientific™ Nunc™ Brank Product, Roskilde, Denmark). In order to assay the ability of *Haemophilus* spp. to biofilm formation, the method based on staining with 0.1% crystal violet described by Christensen et al^37^ and Kaplan and Mulks,^38^ and modified by Kosikowska and Malm^39^ was used. Two hundred microliters of the standardized microbial suspension was inoculated for each well. After incubation time (24 hours, 35°C, 5% CO_2_) measurements of the bacterial growth at OD_570_ were conducted. Then, the medium above the culture was decanted and then the plates were washed extensively 3 or 4 times with distilled water to remove nonadherent or loosely adherent cells, dried in an inverted position and stained with 200 μL 0.1% crystal violet. The plates were left for 10 minutes to stain the cells and washed underdistilled water to remove the unbound dye. Next, 200 μL of decolonizer Color Gram 2 R 3-F (denatured ethyl alcohol–acetone; bioMérieux) was added to each of the wells and the plates were left at room temperature for 15 minutes. Each isolate was tested in triplicate in 3 series. The OD_570_ of the alcohol-dye solution in each well was read. The TSB+HTMS broth without the tested bacteria was incubated under the same conditions and served as blank control. For the purposes of analysis of our results we introduce classification of bacteria as biofilm producers based on optic density (OD) into 5 categories classified on the basis of criteria proposed by Stepanović et al^40^ and modified by Kosikowska.^41^ They defined the cutoff OD (OD_c_) for the microtiter-plate test as 3 standard deviations above the mean OD of the negative control. The bacteria were classified into the categories of biofilm producers as follows: OD ≤ OD_c_—nonproducers (category 0); OD_c_ < OD ≤ 2 × OD_c_—weak producers (category 1); 2 × OD_c_ < OD ≤ 4 × OD_c_—moderate producers (category 2); 4 × OD_c_ < OD ≤ 8 × OD_c_—strong producers (category 3), and the category of biofilm producer added during our studies—8 × OD_c_ < OD—very strong producers (category 4). OD_c_ was 0.12 in our experiments. All tests were carried out 3 times and the results were averaged.

### Statistical Analysis

Data processing and analysis were performed using STATISTICA 2010 Data Analysis Software System (StatSoft, Inc., Tulusa, USA). Frequencies of isolation of bacteria in the follow-up were compared with those at surgery by means of chi-squared test. Relative risk (RR) and their 95% confidence intervals (CIs) were calculated. A *P*-value ≤0.05 was considered as statistically significant.

## RESULTS

**FIGURE 1:**
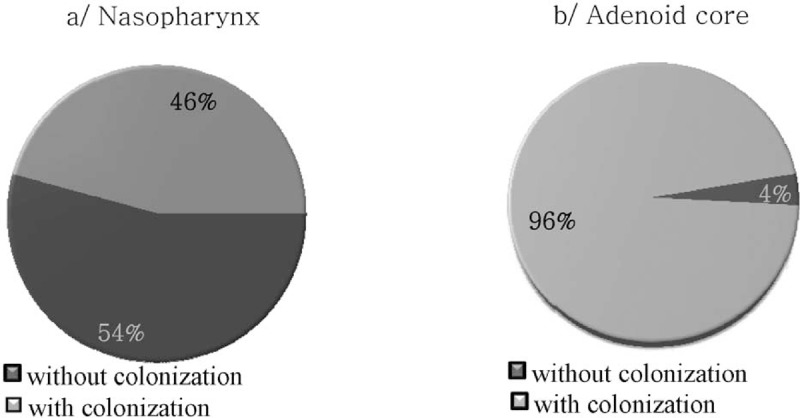
The rate of the nasopharynx (A) and the adenoid core (B) colonization by haemophili in children undergoing adenoidectomy.

According to our results, 159/164 (97%) children who underwent adenoidectomy were colonized by *Haemophilus* spp. The differences in the frequency of haemophili colonization were observed between the nasopharynx and the adenoid core (Figure 1). The adenoid core was colonized in 158/159 (99.4%) children, whereas the nasopharynx in 75/159 (47.2%) children. The differences were statistically significant (*P* < 0.0001, RR = 2.910, 95% CI = 2.398–3.532). The nasopharynx in 51/68 (75%) healthy children was colonized by *Haemophilus* spp. The differences in the frequency of nasopharynx colonization by haemophili in healthy children and in children who underwent adenoidectomy were statistically significant (*P* < 0.0001, RR = 0.396, 95% CI = 0.244–0.643).

According to Table 1, in the group of children who underwent adenoidectomy haemophili, especially *H*. *influenzae* and *H. parainfluenzae*, colonized preferentially the adenoid core or the adenoid core together with the nasopharynx. No colonization of the nasopharynx alone by haemophili was found, except for 1 child's nasopharynx which was colonized by *H*. *influenzae* and for 1 child‘s nasopharynx which was colonized by *H. parainfluenzae*. In total, 120/159 (75.5%) children were colonized by *H*. *influenzae*, and it was the main species isolated from the adenoid core only and both from the nasopharynx and the adenoid core. In 36/159 (22.6%) children *H*. *influenzae* was isolated together with *H. parainfluenzae* and/or other *Haemophilus* species.

**TABLE 1:**
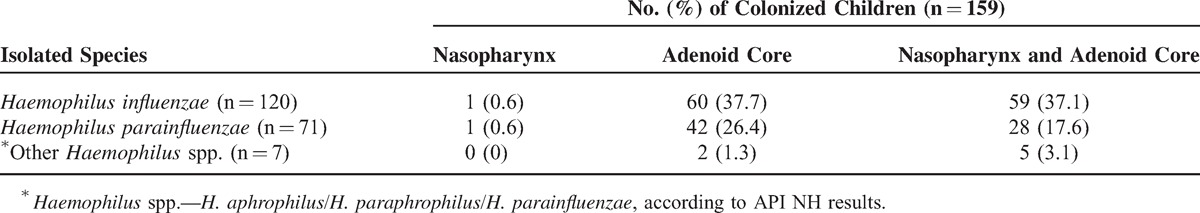
The Prevalence of *Haemophilus influenzae*, *Haemophilus parainfluenzae*, and Other *Haemophilus* spp. Colonizing the Nasopharynx and the Adenoid Core in Children Undergoing Adenoidectomy

The nasopharynx in 20/68 (29.4%) children was colonized by *H*. *influenzae*, in 47/68 (69.1%) by *H. parainfluenzae* and in 5/68 (7.4%) by *Haemophilus* spp. In 1/68 (1.5%) child *H*. *influenzae* was isolated alone, and in 19/68 (27.9%) children *H*. *influenzae* was isolated together with *H. parainfluenzae* and/or other *Haemophilus* species. In 15/68 (22.1%) healthy children from 2 to 3 phenotypically different isolates of *H. parainfluenzae* were selected in each single child. The statistically significant differences in the frequency of *H. parainfluenzae* but not *H*. *influenzae* nasopharynx colonization in healthy children and in children who underwent adenoidectomy (*P* < 0.0001, RR = 0.137, 95% CI = 0.077–0.242 and *P* = 0.638, RR = 1.13, 95% CI = 0.711–1.8, respectively) were observed.

Table 2 shows the colonization of the adenoid core and/or the nasopharynx in children undergoing adenoidectomy by different types or serotypes of *H*. *influenzae*. In 32/159 (20.1%) children 2 or 3 phenotypically different isolates of the same species (*H*. *influenzae* or *H. parainf luenzae*) or serotypes (*H*. *influenzae*) were identified in 1 child. In total, in 65/120 (54.2%) children encapsulated (typeable) *H*. *influenzae* and in 55/120 (45.8%) children NTHi (nontypeable) strains alone and/or in mixed cultures were detected. In 49/120 (40.8%) children only encapsulated isolates of *H*. *influenzae* were identified, in 36/120 (30%) children only NTHi isolates were present, while 12/120 (10%) children were colonized by both types. In addition, 23/120 (19.2%) children were colonized by untypeable (rough—R) *H*. *influenzae* isolates solely or in mixed cultures. In 35/120 (29.2%) children *H*. *influenzae* serotype d was isolated alone or in mixed culture. The adenoid core alone was colonized mainly by a single serotype of *H*. *influenzae*, whereas in case of colonization of the adenoid core together with the nasopharynx multiple serotypes were identified more frequently. The nasopharynx of 18/20 (90%) healthy children was colonized by *H*. *influenzae* strains positive to the poly serum but negative to the b serum (non-Hib). Moreover, 2/20 (10%) healthy children were colonized by untypeable (R) strains.

**TABLE 2:**
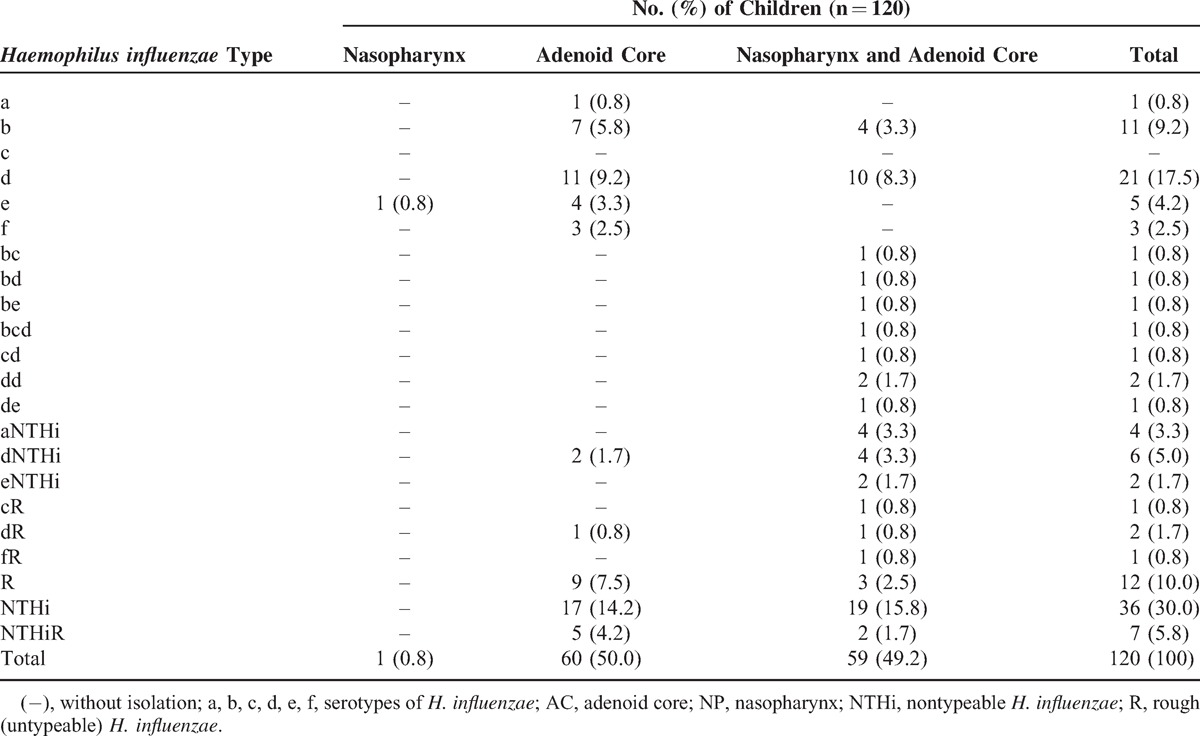
The Frequency of the Nasopharynx and the Adenoid Core Colonization by *Haemophilus influenzae* Types in Children Undergoing Adenoidectomy

In 328 samples obtained from the group of children who underwent adenoidectomy, 302 haemophili isolates were cultured totally. As shown in Table 3, 192/302 (63.6%) isolates were identified as *H*. *influenzae*, 96/302 (31.8%) as *H. parainfluenzae* and 14/302 (4.6%) as other *Haemophilus* spp. (*H*. *aphrophilus*/*H. paraphrophilus*/*H. parainfluenzae*, according to API NH results). *H*. *influenzae* was the most frequently found microorganism in the nasopharynx and in the adenoid core. A total of 90/192 (46.9%) *H. influenzae* isolates were found to be encapsulated and were identified as a–f serotypes. Serotype d was detected with the highest frequency both in the nasopharynx and in the adenoid core. In addition, 77/192 (40.1%) isolates were identified as NTHi and 25/192 (13%) isolates were found to be rough and untypeable.

**TABLE 3:**
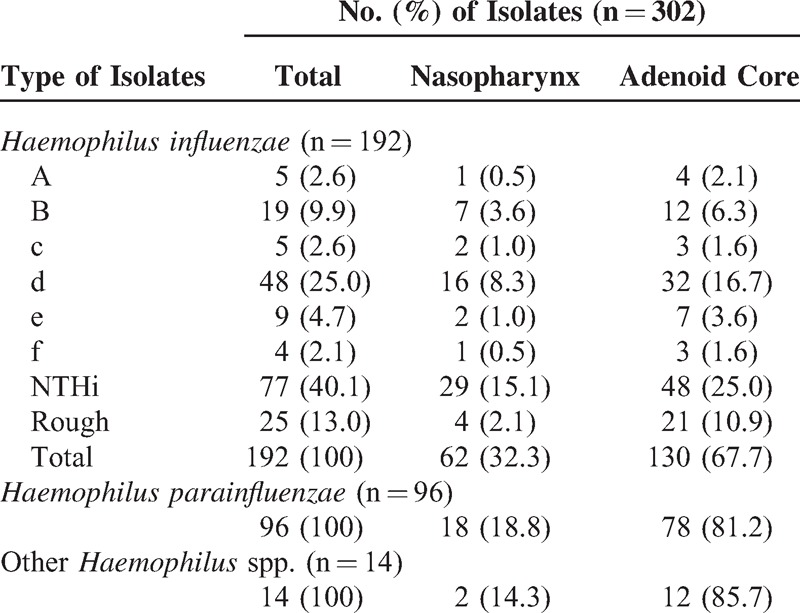
The Prevalence of Haemophili isolates in the Nasopharynx and the Adenoid Core in Children Undergoing Adenoidectomy

In 51 nasopharyngeal samples obtained from healthy children, 70 haemophili isolates were cultured totally: 22/70 (31.4%) isolates were identified as *H*. *influenzae*, 45/70 (64.3%) as *H. parainfluenzae* and 3/70 (4.3%) as other *Haemophilus* spp. *H. parainfluenzae* was the main species isolated from the nasopharynx.

**FIGURE 2:**
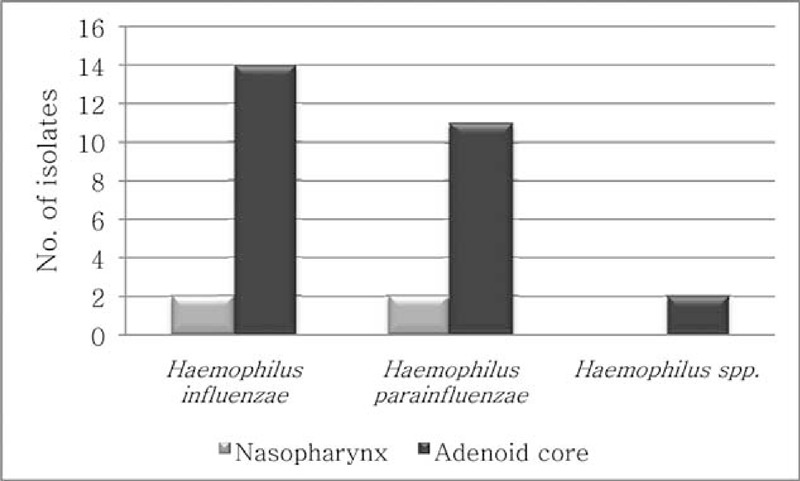
Beta-lactamase production among haemophilisolated from the nasopharynx and adenoid core samples in children undergoing adenoidectomy.

Among haemophili isolates colonizing the nasopharynx and/or adenoids in children undergoing adenoidectomy, 31/302 (10.3%) strains were identified as ampicillin-resistant and beta-lactamase positive—16/192 (8.3%) *H*. *influenzae*,13/96 (13.5%) *H. parainfluenzae* and 2/14 (14.3%) other *Haemophilus* spp. isolates (Figure 2). Beta-lactamase producing haemophili occurred more frequently among the isolates from the adenoid core (12.3%) than those from the nasopharynx (4.9%). However, the differences were not statistically significant (*P* = 0.0859). All ampicillin-resistant haemophili—8/70 (10%) isolates colonizing the nasopharynx of healthy children—were beta-lactamase positive: 4/22 (18.2%) identified as *H*. *influenzae*, 3/45 (6.7%) as *H. parainfluenzae*, and 1/3 (33.3%) as other *Haemophilus* spp.

According to Table 4, 195/302 (64.6%) haemophili isolates colonizing the nasopharynx and/or adenoids in children undergoing adenoidectomy were positive for biofilm production—129/192 (67.2*%)* isolates of *H*. *influenzae,* 54/96 (56.3%) isolates of *H. parainfluenzae*, and 12/14 (85.7%) isolates of other *Haemophilus* spp. were found as belonging to various categories from weak to very strong biofilm producers. The ability to biofilm production was higher in isolates from the adenoid core (141/220; 64.1%) than in isolates from the nasopharynx (54/82; 66%), but the difference was not statistically significant (*P* = 0.8925). Statistically significant differences (*P* = 0.0029) among *H. parainfluenzae* biofilm producers and nonproducers in the adenoid core and the nasopharynx were detected.

**TABLE 4:**
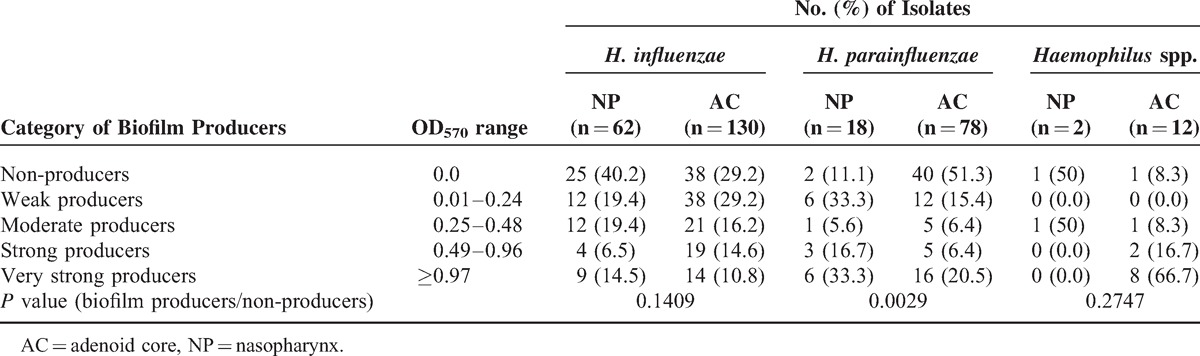
Biofilm Formation by *Haemophilus influenzae*, *Haemophilus parainfluenzae* and Other *Haemophilus* spp. Isolated From the NP or the AC in Children Undergoing Adenoidectomy

**FIGURE 3:**
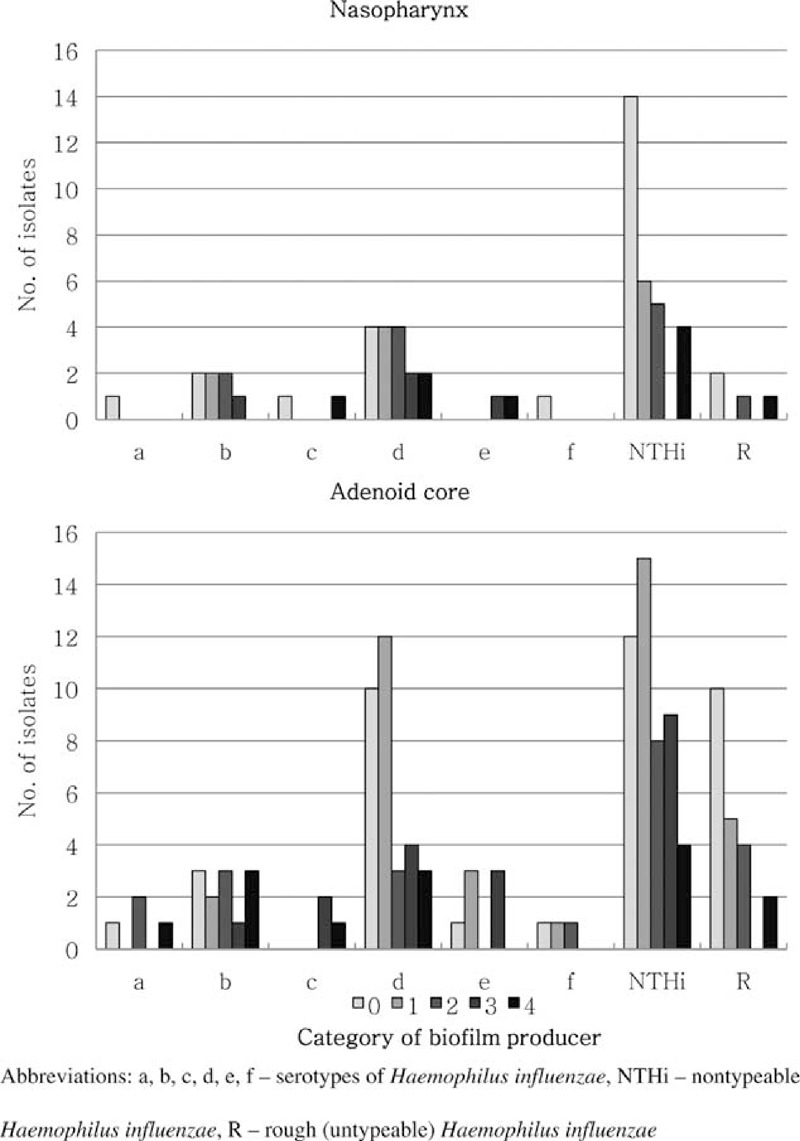
The rate of biofilm producer categories in *Haemophilus influenzae* types isolated from the nasopharynx and the adenoid core in children undergoing adenoidectomy.

As presented in Figure 3, the majority of *H*. *influenzae* isolates possessing the ability to produce biofilm were found to belong to serotype d (*P* = 0.5971) and NTHi (*P* = 1.000), especially those isolated from the adenoid core. Among them the weak producers (category 1) were identified more frequently.

## DISCUSSION

According to our results, both *H*. *influenzae* and *H. parainfluenzae* were isolated in approximately 73% and 43% of children undergoing adenoidectomy, respectively, mainly from the adenoid core. The strong association between the nasopharynx and the adenoid core colonization by *H. influenzae* and *H. parainfluenzae* may suggest the involvement of both species in chronic adenoiditis. This may be confirmed by significant differences between the prevalence of these bacteria in the nasopharynx of healthy children suffering only from mild nasopharyngeal infections and children who need to be adenoidectomized. According to Nistico et al,^42^ adenoids may act as a reservoir of pathogenic bacteria, especially if they are living in the biofilm structure allowing them to persist. Although haemophili, especially *H. influenzae* and *H. parainfluenzae*, are most commonly associated with asymptomatic upper respiratory tract colonization they may also be involved in several diseases.^43–48^ According to the data presented by Korona-Głowniak et al,^36^*Streptococcus pneumoniae* or *Staphylococcus aureus,* apart from *H*. *influenzae,* were frequently isolated in nasopharyngeal and adenoid samples from children who underwent adenoidectomy for recurrent upper respiratory tract infections. These respiratory pathogens highly associated with colonization of the adenoids may use pharyngeal lymphoid tissues as a niche to persist in the host. Although the adenoids and the nasopharynx appear to differ substantially in colonization by pathogenic bacteria, the occurrence of *H. influenzae* and *S. pneumoniae* in the nasopharynx could be predictive of the upper respiratory tract infections.

*H. parainfluenzae* may be an etiologic agent of infections with low potential for virulence.^49^ The low pathogenicity and rare presence of *H. parainfluenzae* may be underestimated because it is treated as a typical commensal or microbial pollution, and because of the problem with its isolation and identification.^44,50,51^ In addition, clinical symptoms of some chronic or recurrent infections by *H*. *influenzae*, especially NTHi, and by *H. parainfluenzae* are very similar.^17,25,27,29^ A growing number of *H. parainfluenzae* infections were detected in young children and newborn or preterm infants.^23,52,53^ In pediatric patients primarily diseases of the upper and lower respiratory tract, endocarditis and meningitis are particularly relevant.

Other authors observed that the rate of *H*. *influenzae* colonization in children > 1-year-old can range from approximately 27% to as much as 90%, depending on the type of diagnostic materials and methods or other risk factor.^2,54^ In addition, the microbial colonization may be influenced by several factors, such as the age and clinical condition of children, individual sensitivity of mucous membranes, the degree of exposure to the bacteria, contacts with other children, the socioeconomic conditions, behavior and habits of parents, passive smoking, number of siblings, and the race.^36,47,55^

Data presented in this article showed that in case of colonization of the adenoid core together with the nasopharynx multiple serotypes were identified frequently. Among children colonized by *H*. *influenzae* in 1 child 1 to 3 different serotypes and/or untypeable strains were isolated, mainly from the adenoid core. It was possible because of detail analysis of 1 sample in regard to a lot of morphological different colonies. Encapsulated (typeable) strains *H*. *influenzae* were isolated in approximately 54% of children, whereas NTHi were isolated in approximately 40% of children. The detection of different *H*. *influenzae* types and/or other haemophili in 1 child both in the nasopharynx and in the adenoid core, suggests important role of these microorganisms in children with recurrent pharyngotonsilitis.

According to literature, the upper respiratory tract of 20% to 96% children was colonized mainly by NTHi.^15,35^ The biggest influence on the distribution of capsular serotypes and NTHi in the pediatric population was exerted by the implementation of universal compulsory vaccination of infants against *H*. *influenzae* type b—Hib.^8,56,57^ The introduction of the Hib conjugate vaccine into national childhood immunization programs has resulted in a reduction of Hib diseases in many countries.^9^ However, now it is possible that other *H*. *influenzae* strains (non-b serotypes or NTHi) or other opportunistic pathogens could take the place of serotype b, especially in older children and adults.^8,56^ It is reported that most *H*. *influenzae* colonizing the upper respiratory tract are noncapsulated and data from the present study shows the opposite. A lot of typeable strains including serotype d were detected during ours examination. In our point of view these results are new and different from those have been described by other authors. In 1 child usually the presence of 1 serotype of *H*. *influenzae* was demonstrated.^58^

According to Skoczyńska et al,^59^ approximately 6% of *H*. *influenzae* isolates obtained in Poland from patients with lower respiratory tract infections were found to be encapsulated; the most common were serotypes b (40.3%) and e (38.9%) followed by serotypes f (16.7%) and d (4.1%). In our studies, 35 (29.2%) children were colonized by serotype d. *H*. *influenzae* serotype d was isolated from both the cerebrospinal fluid and the blood of adults with meningitis.^60^ Moreover, it was an etiological agent of meningitis in infant,^4^ maternal and neonatal sepsis,^61^ mild respiratory disease in neonates,^53^ of pneumonia, empyema, and shock.^6^ According to Sakano et al,^62^ this type of bacteria may be an important causative agent in immunodeficient patients. It is possible that the diagnostic accuracy of serotype identification by commercially available slide agglutination serotyping tests was partially limited in false-positive results and differed among the laboratories. For this reason, in many laboratories a polymerase chain reaction (PCR) procedure, which is available in specialized laboratories, was performed to detect genes specific for capsule types a—f and NTHi species.^63,64^ Discrepancy between the results of antigenic examination and PCR method was found by Falla et al,^65^ LaClaire et al,^66^ and Luong et al.^58^

Our data indicate that approximately 65% of the haemophili from the nasopharynx or adenoids samples could produce biofilm. These results suggest the possible role of *Haemophilus* spp. biofilm in upper respiratory infections, especially in recurrent or chronic infections associated with bacteria persistence, as was suggested by other authors.^32,67–69^ The presence of biofilms was also detected, for example, in endocarditis, chronic and recurring respiratory infections, urinary tract infections, kidney stones, otitis media, tonsillitis, and rhinopharyngitis.^30,31^ The ability of *H*. *influenzae* to biofilm formation and the role of biofilm presence, for example, on the surface of adenoids in children undergoing adenoidectomy and in respiratory infections, was confirmed.^34,70,71^ At the follow-up visits, which were performed during a few months (from 1 to 6) after the adenoidectomy, a decrease in mucosal colonization of the nasopharynx by bacteria with an ability to biofilm formation was observed. The microflora restoration in the nasopharynx in children may indicate that *H*. *influenzae* living on the surface of the adenoids were invasive strains. Biofilm formation by these bacteria was an important factor in the occurrence of recurrent infections not susceptible to the therapy. Subsequent reports on the role of NTHi biofilm colonization of tissues and the pathogenesis of chronic infections in children and adults, such as otitis media, sinusitis, cystic fibrosis, or chronic obstructive pulmonary disease were published.^7,32,70,72^

Our results showed statistically significant differences among *H. parainfluenzae* biofilm producers and nonproducers in the adenoid core and the nasopharynx. There are only fragmentary reports on biofilm formation by *H. parainfluenzae.*^39,41,73,74^ It is known that *H. parainfluenzae* is called the early colonizer and cocreator of multigrade plaque biofilm created by various species of bacteria found in the mouth^75,76^*H. parainfluenzae* is a rather rare pathogen for inflammation within tonsils and adenoids. However, a significant incidence of its colonization especially in the adenoid core and their ability to biofilm formation may suggest the involvement of this microorganism in chronic upper respiratory tract infections. Moreover, several studies describe biofilms formation by unidentified bacteria on the surfaces and crypts of adenoids removed from children.^42,70,77,78^

Our study presents new results because there is scarce information on the subject of colonization and serotyping of *H*. *influenzae* worldwide. Biofilm production by haemophili, both *H*. *influenzae* and *H. parainfluenzae*, is also an important question that has been studying in these later years. We assume that biofilm may be an important structure for the survival of these bacteria and in the early phase of infection. We conclude that the haemophili biofilm both in the nasopharynx and in the adenoids allows them to persist in the host and could prefer the survival of pathogenic or opportunistic species. On the basis of literature and data presented in this article, we agree with the statement that the incidence of *H. influenzae* isolates associated with chronic or invasive infections should be monitored and notified as a way to evaluate the changes and trends in these bacteria serotype or nontypeable strains distribution. Moreover, the confirmed and increased cases of infections (even life-threatening ones) caused by *H. parainfluenzae*, especially in very young children, allowed us to postulate extensive studies on this species and its involvement in the human pathology, especially in recurrent or chronic upper respiratory tract infections, including adenoiditis.

## CONCLUSIONS

*H*. *influenzae* and *H. parainfluenzae* carriage rate was comparatively higher in the adenoid core than that in the nasopharynxin children undergoing adenoidectomy, suggesting their involvement in chronic adenoiditis. The growth in the biofilm seems to be an important feature of haemophili colonizing the upper respiratory tract responsible for their persistence.
